# Exploring Consumer Experiences of Barriers and Enablers to Accessing Rehabilitation That Meets Their Needs: The Rehabilitation Choices Study, Part 2—Consumer Perspectives

**DOI:** 10.1111/hex.70035

**Published:** 2024-09-24

**Authors:** Gillian Mason, Karen Ribbons, Lucy Bailey, Adrian O'Malley, Tracy Ward, Stephen Ward, Michael Pollack, Frederick R. Walker, Michael Nilsson, Nicolette Hodyl

**Affiliations:** ^1^ Hunter Medical Research Institute New Lambton Heights New South Wales Australia; ^2^ Centre for Rehab Innovations The University of Newcastle, Australia Callaghan New South Wales Australia; ^3^ College of Health, Medicine and Wellbeing The University of Newcastle, Australia Callaghan New South Wales Australia; ^4^ Hunter New England Area Health Service John Hunter Hospital New Lambton Heights New South Wales Australia; ^5^ Lee Kong Chian School of Medicine Nanyang Technological University Singapore Singapore; ^6^ NSW Regional Health Partners Newcastle New South Wales Australia

**Keywords:** access to healthcare, accessibility, decision‐making, disability, people with disabilities, rehabilitation

## Abstract

**Introduction:**

Improved access to rehabilitation is highlighted as a key pathway to achieving the World Health Organisation's (WHO) goal of ensuring healthy lives and promoting well‐being for all (Sustainable Development Goal 3). This article is the second in a two‐part series outlining the findings from the *Rehabilitation Choices* study, which aimed to identify how health professionals and consumers in Australia are informed to make decisions about rehabilitation, and their experience with barriers and enablers to accessing that rehabilitation. In this study, we present the perspectives of consumers with different health conditions and a range of experiences with rehabilitation services.

**Methods:**

This was a qualitative study using focus groups and semi‐structured interviews. People with self‐reported lived experience of rehabilitation and carers were recruited using maximum variation sampling. Thematic analysis of data was conducted using an inductive approach.

**Results:**

Fifty‐six consumers with diverse lived experiences of rehabilitation (19–80 years, 49 patients, 7 carers) participated in focus groups and interviews to discuss how they sourced information about rehabilitation and their experiences of what made it hard or easy to access rehabilitative care to meet their needs. Four themes were produced from the data: (1) service‐centricity of options limits access, (2) access is the patient's responsibility, (3) enabling decision‐making about rehabilitation with appropriate information and (4) provision of a psychologically safe environment.

**Conclusions:**

Any planned (re)design of services to improve consumer access to rehabilitation should consider the themes identified in this study. This will ensure that consumers are provided with rehabilitation options that suit their holistic and unique needs beyond consideration of their medical diagnoses, and are actively supported to navigate this access, provided with information to help them make informed choices and provided a psychologically safe environment to engage effectively with rehabilitation.

**Patient or Public Contribution:**

Three consumer research partners with lived experience of rehabilitation as patients or carers were core team members. They were involved in the design and implementation of the recruitment and communications strategies, design of the interview approach and discussion guide, contributed to the interpretation and contextualisation of findings and writing of this manuscript and are included as co‐authors (A. O., T. W. and S. W.).

## 1 Introduction

Rehabilitation medicine is defined by the World Health Organisation (WHO) as the interventions designed to optimise functioning and reduce disability in individuals with health conditions in interaction with their environment [1, 2]. Rehabilitation is about more than improving physical health: it aims to optimise a person's ability to think, see, hear, eat or move around so they are better able to participate in activities necessary and important to them [2]. According to the WHO [2], one in three people globally live with a condition that would benefit from rehabilitation [2]. Universal access to rehabilitation has been acknowledged as key to the achievement of Sustainable Development Goal 3, which aims to ensure healthy lives and promote well‐being for all at all ages, as well as an essential part of achieving target 3.8, which focuses on achieving universal health coverage, including financial risk protection, access to quality essential health‐care services and access to safe, effective, quality and affordable essential medicines and vaccines for all [3].

Within the context of healthcare in Australia, rehabilitation is conceptualised as both an intervention and a service [4]. The term ‘consumer’ refers to those who use, have used or are potential users of health services, including patients, carers, friends and family members with a lived or living experience in specific health area. In Australia, compared to healthcare service provision in the United States (predominantly private system) and the United Kingdom (predominantly public system), the system is a hybrid of public and private medical and allied health services. In 2016, 451 rehabilitation medicine specialist doctors were registered, providing 1.8 clinicians per 100,000 population, of whom 37.7% were employed in the private sector [5]. Most rehabilitation services comprise a multi(inter‐) disciplinary team, often led by a qualified rehabilitation medicine physician within both the private and public healthcare sectors. Services are provided across a range of settings from hospital to community‐based settings provided as in‐reach, inpatient and outreach programmes, with ambulatory care services provided in the outpatient, day hospital and home‐based locations [4]. In 2015–2016, almost 435,000 discharges were reported for in‐patient rehabilitation care across Australia, with 76% occurring in private hospitals, and about 80% were for people over the age of 60 years [6]. The number of community‐based or self‐referrals to rehabilitation, and the number of people needing but unable to access rehabilitation, is unknown.

Available evidence suggests that the decisions people make about the rehabilitation activities and services they seek to engage with are influenced by their health providers instructions, advice and support with direct referrals [7, 8, 9], and also the opinions of their family, friends and their own research [10]. Beyond receiving a referral to rehabilitation, whether patients attend or continue with their rehabilitation is influenced by several factors relating to accessibility. Characteristics of accessibility include services being approachable, acceptable, available, affordable and appropriate, as well as factors relevant to patients—the ability to perceive, seek, reach, pay and engage with services [11]. Ensuring services are accessible is an international issue; for example, even in high‐income countries, access to inpatient rehabilitation for stroke patients who need it is poor (13%–57%) [12].

The *Rehabilitation Choices* study was conducted to explore decision‐making around engagement with rehabilitation approaches and programmes and to identify the enablers and barriers to accessing services that meet the needs of consumers in the Australian context. Specifically, this study explored the following research questions: (1) ‘How have consumers of rehabilitation been informed about the rehabilitation they need?’ and (2) ‘Which factors act as enablers and barriers for consumers to access the rehabilitation they need?’

## 2 Materials and Methods

### 2.1 Study Design

This was a qualitative study using focus groups and semi‐structured interviews. Care was taken to design the study to be as broadly accessible and relevant as possible to a diverse cohort. The recruitment strategy, all public‐ and participant‐facing study communications, the consent process and the discussion guide were co‐designed by the research team, which included three consumer representative members. Ethics approval was granted by the University of Newcastle Human Research Ethics Committee (approval H‐2020‐0324) on 4 November 2020.

#### 2.1.1 Eligibility, Recruitment, Screening and Consent

Recruitment was via (1) social media posts on Facebook, X (formerly Twitter) and LinkedIn from the accounts of the Centre for Rehabilitation Innovations, Hunter Medical Research Institute (HMRI), University of Newcastle, Stroke Foundation and by researchers on their own pages, in a number of Facebook groups for people with chronic disease and recent surgery; (2) by word‐of‐mouth; and (3) by direct invitations to participants of a concurrent research project that involved people who had total knee replacement surgery between 1 month and 1 year prior. Interested people were sent a participant information sheet and consent form and invited to discuss the study with the research team by phone, video call or email before giving consent to participate. Some participants chose to take part with a carer to assist them with communication or because the participant wanted the carer to share their own views about the rehabilitation experience. When the carer participated to share their own views, they were included as study participants. Inclusion criteria included: (1) over 18 years of age; (2) self‐report of current or previous engagement with rehabilitative care, or support of someone in their rehabilitation as a carer or care partner; (3) having lived in the community; (4) willing to participate in a focus group or 1:1 interview via videoconference (due to the COVID‐19 restrictions at the time of study). Exclusion criteria included those who: (1) had not participated in rehabilitation, (2) were still receiving inpatient care and (3) lived in a residential aged care setting. We used a maximum variation sampling strategy [13, 14] to capture a broad range of experiences by monitoring participants' characteristics (health condition, injury, disability, other factors contributing to the need for rehabilitation) during recruitment. We sought to include participants who were broadly representative of varying age, gender, diagnoses, disability status or identity, rurality and time since, length of and mode of rehabilitative care. This enabled us to explore those barriers and enablers to rehabilitation that were common across a number of conditions. Informed consent was obtained from all participants before study participation.

### 2.2 Data Collection

Data were collected between February and June 2021 via focus groups (*n* = 36, 60–90 min in duration) and 1:1 semistructured interviews (*n* = 20, 20–30 min in duration). Facilitation was by members of the research team with experience in qualitative methods and disability inclusion (N.H. [female, PhD] and G.M. [female, BPhysio]) via videoconferencing technology (Zoom). The transcription function on the videoconferencing app was used to transcribe focus groups, whereas notes were taken by the facilitators during interviews. These were shared with participants to ensure accuracy before analysis.

#### 2.2.1 Focus Group and Interview Preparation

Participants were contacted before the focus group or interview to understand any access requirements, including provision of aphasia‐friendly and easy English written materials with visuographic supports, allowing further reflections after the focus group to be submitted by email, inclusion of people who didn't have access to a device or the Internet at home by facilitating a connection from a relative's or friend's house, participating with the assistance of a care partner and/or choosing a 1:1 semistructured interview instead of focus group for fatigue, cognitive overload or scheduling reasons. A link to a short video on YouTube that explained the videoconference functions and expectations for the session was sent by SMS to participants at the time of booking, and then videoconference and device test/setup or training sessions were offered by the research team to support participation.

Before the focus groups and interviews, each participant received a ‘Chat‐in‐a‐Box’ package by mail, adapted with permission from the *Workshop‐in‐a‐Box* approach used by Heiss [15]. This approach was taken to create a sense of welcome and hospitality and a shared experience for participants in the absence of being able to meet in person due to public health measures in place during the pandemic. The package contained a welcome letter with general instructions, a sweet treat, tea and coffee bags and coloured paper each containing different topics for discussion that participants could use (optional) to prepare for focus groups if they wished, as a memory aid or to support attention and communication difficulties. During discussions and interviews, reference was made to these topic sheets as a cue for discussion.

The topics for discussion were as follows:

1. Who gave you advice about rehabilitation (people who informed you)?
2. Where else did you get information (how you informed yourself, resources, research)?
3. What made it hard to get the rehabilitation you needed (hurdles)?
4. What made it easy to get the rehabilitation you needed (green lights)?

### 2.3 Data Analysis

The facilitating investigators (N.H. [an academic researcher] and G.M. [a clinician‐researcher with experience as a rehabilitation provider and service manager], both of whom also had personal lived experience of participating in rehabilitative care) led the data analysis. To establish positionality and reflect on potential researcher bias, they met before data collection commenced to discuss and document their own professional and personal experiences of rehabilitation. These notes were used for reflection during data analysis. Three investigators led the thematic analysis [16], independently reading and coding data from the transcripts and meeting notes. These were discussed and collated into candidate categories and themes using an inductive approach [16], with ongoing reflexive dialogue with team members to ensure that their own experiences did not affect the interpretation of the data.

Themes were developed through an iterative process according to each of the research questions, with input from the three consumer representative team members (A.O., T.W. and S.W.) and, by consensus, merged into final themes. Preliminary results were then presented back to all research participants, who were given an opportunity to challenge and make suggestions about themes and add to their contextualisation. These were then further refined by combining the themes derived from each of the research questions to identify common themes underlying access to rehabilitation.

## 3 Results

### 3.1 Participant Characteristics

Forty‐nine people with lived experience as patients and seven people who identified as carers or care partners (19–80 years) expressed interest in participating in the study; all were enrolled, and no participants chose to withdraw. Although participants cited a diverse range of diagnoses, all participants chose a key health condition to cite as the reason they needed rehabilitation (Table 1), but many noted that their functional impairments and rehabilitation needs were the result of the intersection between these, comorbid conditions and other factors.

**Table 1:** Key health conditions (diagnosis) cited by participants as the reason for the rehabilitative care they engaged with.

Key condition	Patients	Carers/care partners
Total knee replacement (TKR)	24	1
Stroke	12	5
Back pain	2	
Total hip replacement (THR)	1	
Traumatic brain injury (TBI)	1	1
Chronic inflammatory demyelinating neuropathy	1	
Lung transplant	1	
Cancer	1	
Fractured femurs	1	
Fractured wrist	1	
Cardiac event	1	
Bilateral ankle fractures	1	
Interstitial lung disease	1	
Ehlers's Danlos Syndrome	1	
Total (*n* = 56)	49	7

Participants engaged with rehabilitative services and programmes across a range of public and privately funded health settings, including in hospitals (some in specialised rehabilitation units and others in standard wards), as outpatients via a day hospital programme, in a series of appointments at a hospital‐based clinic, in the community (i.e., at a health professional's private rooms or a community health centre) or in their own home. Participants had been provided care by health professionals who were individuals or part of multidisciplinary teams over a range of timeframes (ranging from 2 weeks to several years), and on a 1:1 or mixed basis, via various modes including in‐person sessions, telehealth (real‐time phone or video calls), asynchronous messaging with remote monitoring or participation in exercise programmes delivered and supervised via mobile health applications (mHealth apps) accessed on smartphones or smartwatches or via hybrid models of care.

### 3.2 Accessing Rehabilitation

Four themes were identified relating to the receipt of information and the barriers and enablers to accessing rehabilitation: (1) service‐centricity of options limits access, (2) access is the patient's responsibility, (3) enabling decision‐making about rehabilitation with the appropriate information and (4) provision of a psychologically safe environment. Supporting quotations for each theme are provided in the text and the Supplementary Material, showing participant citation details including the participant study identifier number, age, sex and condition.

### 3.3 Theme 1: Service‐Centricity of Options Limits Access

Participants felt like rehabilitation was offered as a standard pathway or package of care with a ‘one‐size‐fits all’ approach that wasn't offered as being tailorable:

*… text book’ approach by staff—they didn't expect that people's needs or progress might fall outside the course that was expected … they weren't trying to understand her, they were just fitting her in around the same model they do for everybody*. (ID13, 70 years, m, carer of survivor of stroke)

This rigidity of services created and compounded access and equity issues for participants. Those who had complex or rare conditions, disability access needs, transport issues, technology and Internet availability challenges, family or caring responsibilities, cultural needs outside the dominant paradigm or atypical clinical presentations or personal goals felt uncomfortable speaking up to ask staff for ‘special’ treatment. ‘*We can't do any more for you, we're used to having older people*’ (ID50, 46 years, f, cardiac).

Participants talked further about rehabilitation appearing to be designed around existing funding mechanisms and in accordance with policies and guidelines that mandated specific time windows for access. Some consumers reported not being psychologically ready for rehabilitation at the time when it was offered. ‘*… huge pressure to do rehab immediately, around therapists’/service's timetable above all else, irrespective of fatigue or psychological readiness*’ (ID13, 70 years, m, carer of survivor of stroke). Some were then unable to access it later when they felt ready, or it was extremely difficult to get access to it later.

*I was still in denial about this actually happening to me, [so did not take up what was offered] … I think once you're out of denial and actually go “Okay, this has happened, and now what am I going to do about it?”, that's when you need to be able to access rehab*. (ID5, 42 years, f, stroke)

Being offered flexibility, like the ability to book sessions or being able to set a schedule in advance to ‘*… be able to plan life around it*’(ID29, 59 years, m, TKR) was valued and framed as an access and equity issue, rather than merely a convenience issue. Having to fit in with a rehabilitation timetable set around therapist or service availability often caused financial, personal, or family stress and had disproportionate impacts on different people. A parent who was juggling the needs of their children during their rehabilitation reported the impact on them was significant ‘… *you couldn't choose the time, and when you are needing people to drive you there and you've got people driving, you know, your kids to school that you might have normally done … There is a lot of arranging you have to do but you have to fit into their schedule*’ (ID50, 46 years, f, cardiac event]. A participant who was retired reported that the key to their ability to participate in what was offered was the free time they had, as they were retired. ‘*I had left work, so I had all this time … I think time was the biggest factor to be able to put this all into place*’ (ID 33, 62 years, TKR).

Local policies designed with good intentions, for example, mandating carer involvement in long‐term rehabilitation programmes to encourage adherence, created hardship for consumers who lived far away from the rehabilitation centre or who were faced with a binary choice between missing out on rehab entirely or forfeiting the sole family income for extended periods. Also, those juggling other responsibilities, some consumers who said they valued autonomy, describing themselves as ‘*self‐directed*’, ‘*proactive*’ or ‘*motivated*’, found it preferable to be given some guidance before being left to work independently, or supported by mHealth apps.

The rigidity of service offerings was also described in the context of the availability of services being tied to specific settings and modes of delivery, rather than accessible in ways that might work best for the patients. One participant talked about needing multidisciplinary support with rehabilitation that was available as an inpatient; however, when they needed to leave the hospital for mental health reasons, none of the care was accessible to them in other modes (e.g., as an out‐patient or in the home). Others talked to examples of services being ill‐equipped to provide tailored care even when it was clinically needed. ‘*Group rehab focusses on the weakest link, not the needs of all*’ (ID32, 66 years, m, TKR).

Participants reported a general lack of recognition about the need for inclusion of cognitive and psychological interventions in rehabilitative care unless they specifically met the criteria for psychiatric rehabilitation. Reports were of an almost universal absence of psychologists on the rehabilitation teams with which participants had engaged. Participants concurred that needing rehabilitation was inherently stressful and that interventions to support mental health should be considered part of a standard suite of interventions in medical rehabilitation, rather than being reserved as a ‘rescue’ measure.

*There needs to more availability of psychological support. It's not a disgrace to be not coping, but you're made to feel like that. And trying to access appropriate sorts of support, it just isn't out there*. (ID2, 67 years, f, stroke)

### 3.4 Theme 2: Access Is the Patient's Responsibility

Participants reported a pervasive lack of assistance with navigation to, within and between disconnected services and programmes. They reported feeling like providers did not share responsibility in the process of a patient securing access to the rehabilitation they needed, and that such support would be helpful. ‘*The onus is on you*’ (ID4, 48 years, f, chronic inflammatory demyelinating polyneuropathy). Lack of timely access to straightforward, digestible information about processes and an understanding of the varying roles and responsibilities of different professionals within the system made it difficult to understand who was accountable for actions and sharing information. This was a common experience for consumers, including those who had a high level of general knowledge or even had experience working in the health system.

*I rang the hospital and said, ‘What happened to the physiotherapist, or the rehab?’ And they said, ‘Oh, you didn't book it before you left hospital, it's got nothing to do with us now*’. (ID37, 76 years, f, carer of person who had TKR)

Participants reported feeling that their rehabilitation providers expected their patients to ‘take responsibility for their own recovery’, requiring them to deprioritise other life responsibilities to participate in the set programme and absorb whatever costs or impacts this might incur. Some participants described needing to take multiple days off paid work to attend face‐to‐face or all‐day programmes. Having options for therapy delivered in‐person at a centre, remotely, or in the home, via telehealth, virtual care and hybrid options radically improved access for consumers, particularly those who had long‐term rehabilitation needs.

*The logistics of you know, just getting yourself to an appointment for an assessment (that could have been by telehealth), when you can barely walk you know 10 or 15 meters. Catching a two‐hour train trip to see a specialist and he gets you in and out in five minutes and you think, 'well what the hell was that all about?'. And then you come home in pain*. (ID3, 47 years, m, back pain)

Participants noted that when providers set up telehealth and virtual care approaches without clear communication processes, adequate support or consideration for the preferences, skills or capacity of the participant, access could be difficult but it was the responsibility of the patient to work through this on their own. ‘*There have been occasions where my level of distress has escalated massively because I'm battling with technology …Telehealth is fantastic, but I sometimes think that due diligence about the user's capacity isn't always taken into account*’ (ID21, 61 years, f, stroke). Similarly, participants described being tasked with compulsory, multistep processes and complex forms required to gain access to rehabilitation:

*You have to get your GP to sign off on it, and if they don't fill the form out correctly, then you're back there five different times and … when our energy is a major commodity of ours it's so valuable. You're using all this energy on non‐productive things*. (ID52, 61 years, m, stroke)

Online information and forms were reported as not being accessible to those who were not technology literate or those who did not have independent access to the Internet. Participants with communication disabilities reported being unable to find information in accessible formats and shared their experience of not being invited to share their access needs. It felt like their responsibility to adapt, advocate or engage a carer to help them, rather than having access to processes and care that was accessible by design, or because the service had adapted it for them.

### 3.5 Theme 3: Enabling Decision‐Making About Rehabilitation With Appropriate Information

Participants expressed a strong desire to have access to information to support choices about their rehabilitation. This ranged from information about what options they had, what services exist that may be helpful, what the therapy could or would entail, whether criteria exist to access different services, what parts of the rehabilitation were customizable versus stringent offerings and whether other patients engaging in this rehabilitation are ‘like them’. They noted that this information would help them understand why different referrals were made, whether they would feel safe to engage and what options they had relating to their own care.

Participants who were provided with the choice between various programmes, services or settings (e.g., a time‐bound inpatient or day hospital programme) reported being given limited information to truly understand what their choice would mean in terms of activities they could engage in and the impact that participation in the different programmes might have on their lives. This lack of information made it difficult for consumers to weigh up the costs and benefits of different programmes and discuss this with providers.

*There should be better explanations as to why rehab is planned how it is. I know it's based on science and the availability of the rehab services and providers, but if you understand it more, it's easier to engage and ask for modifications if needed*. (ID51, 61 years, f, cancer)

Participants reported wanting more information about options that might involve less intensive health professional involvement, were available in different settings and at which point in their recovery an episode of rehabilitation would be considered ‘finished’.

*… with the rehab, is it if I get up, grab my dogs and walk briskly up [name of] St, and then come home and do some work with my elastic straps and then go and have a swim … am I just enjoying my life or am I still doing rehab?* (ID32, 66 years, TKR)

Clear, timely, accessible, and specific communication of information increased participants' perceived ease of access to services and their ability to make informed decisions. This was seen as key to effective planning, joint goal setting, achieving optimal participation in rehab and managing expectations. Participants expressed great value in information that accommodated their individual communication or cognitive impairments and catered for individual preferences and capacity (e.g., recognising differences in health literacy, offering multimedia options and varying the level of detail), including the involvement of a carer where required. ‘… *it makes it so much easier because you get someone like [patient] to whom you say "Oh, and what was said there?" and he says "Ah, I don't remember"*’ (ID43, 68 years, f, carer of person who had TKR). This information needed to be communicated in a way that met the needs of the person. ‘*… for people who don't understand the system or can't communicate quickly or need accommodations to communicate … if you can't understand and communicate quickly, you can't be included in the planning*’ (ID21, 61 years, f, stroke).

Participants reported often receiving advice from people other than their health professional. This included information and advice from their family and kinship groups, friends and acquaintances. ‘*There's some advice coming from everywhere. Everyone you meet is like "I know a guy"*’ (ID7, 42 years, m, back pain). Some participants reflected that despite their specialist doctor's advice that they didn't need it, they had entered inpatient or extended outpatient rehabilitation, as this was perceived to be a ‘premium’ service. ‘*I had spoken … to a few past patients … and they spoke so highly about (rehabilitation facility) that I thought I would maybe get a bit more benefit and quicker recovery if I looked into getting into there*’ (ID19, 73 years, m, TKR). Participants considered retrospectively that if they had been shown illustrative data or been provided with more assurance that someone like them did not need such intense rehabilitation, they may have accepted their provider's initial advice. ‘*… a good enough explanation would give me the confidence that I didn't need to do something else, (but) I pursued something else, because I felt like I hadn't received the answers that I had asked for*’ (ID48, 48 years, f, fractured wrist) Participants also reported how they sourced and valued experiential knowledge from people who had the same conditions or experiences, via support groups, online blogs or video content (e.g., YouTube) or Facebook groups. Participants shared that they were well aware that others' experiences and ‘internet advice’ might not be evidence‐based, relevant to or safe for them personally but felt that seeking information from peers should be encouraged, and health professionals should be ready to discuss it, or, at minimum, not be dismissive. ‘*I guess I've gained so much information from them (Facebook groups) and only a little bit from medical professionals*’ (ID4, 48 years, f, chronic inflammatory demyelinating polyneuropathy).

Participants, especially those with more complex situations, reported difficulty finding and synthesising information to determine whether a service had the specific expertise to suit their specific rehabilitation needs. This left participants not knowing whether the rehabilitation would be worth their investment of time, energy and finances.

*I had no idea how to choose, so I was just pointing off the list, like ‘Oh, I'll have that one!’ I was given access to a list of names of people who provide services. But I didn't know [anything about] them, so it was more luck than good management who I got to choose … there wasn't anything that I … could access, to say oh this person's a really good one*. (ID13, 71 years, m, carer of survivor of stroke)

Some participants explained further that static information wasn't sufficient; they would have liked a way to interact with information in a multimodal way, with the opportunity to seek information about whether or not it would be relevant to them personally. Written communications that supplemented a two‐way dialogue between consumer and health professional made things clearer and more straightforward. ‘*Information needs to be in smaller bites, slow it down, pen and paper, listen to the person*’ (ID45, 35 years, f, stroke).

Participants reported a lack of available information relating to the costs of rehabilitation as an impediment to being able to make an informed choice. Participants talked about the mental and emotional energy required to gather, understand and weigh up options against their costs in a timely manner. More and easily accessible information was desired to understand costs, depending on which public services one might be eligible for and which might need to be privately funded. ‘*… financially it's … a bit of a minefield*’ (ID3, 47 years, m, back pain).

### 3.6 Theme 4: Provision of a Psychologically Safe Environment

Consumers reported it feeling easier to engage with rehabilitation when they felt they were in a psychologically safe space. It felt safe when providers were perceived to be genuinely interested in a patient's recovery and showed empathy about the impact their illness or injury may have had on their life as an individual and a member of a family and community. ‘*It's very encouraging if the other person knows your condition … they can relate to you personally and give you the undivided attention. It encourages you that perhaps they do know the condition. And the options available for you*’ (ID52, 61 years, m, stroke).

The creation of a psychologically safe space meant participants felt confident and at liberty to communicate their unique aspirations, concerns, challenges and priorities with providers, especially if they lived with a rare condition or were having an atypical recovery.

*It takes a lot for us to get ourselves, whether it's physically or mentally, to one of these services and so, once we get that we want to feel validated …. to be treated with respect and validation*. (ID4, f, chronic inflammatory demyelinating polyneuropathy)

Being heard, respected and supported to access the available options, even when having made choices that weren't in close alignment with a health professional's recommendations, provided a sense of personal agency and control over one's own recovery, contributing to this feeling of psychological safety. For some, this perception was created by having a trusted person involved as an advocate who could help communicate their needs. Advocates included general practitioners who knew the participant well, family members and, in particular, family members who were also health professionals. Conversely, participants expressed disappointment when the health system did not acknowledge and respect them as whole, intelligent and unique people.

… *it's just an appointment, for them, it's just a thing in their diary. And this (affects how easy it is to engage in rehab) because it's the case that you can't move on, you see the glass half empty, so you think, ‘No. I'm not going to do it’. Recovery depends on the positive or negative experience that you have*. (ID23, 46 years, f, stroke)

Some participants experienced feeling psychologically unsafe when, for example, they felt they were being judged harshly for ‘non‐compliance’ or were perceived as acting ‘entitled’ or ‘difficult’ when seeking flexibility in how they might engage with rehabilitative care offerings. This was especially so where patients had communication needs or disabilities that weren't accommodated, cultural needs or caring or other responsibilities that they felt needed to be prioritised over perfect adherence to a gold‐standard rehabilitation plan.

*I can't imagine what the hospital would do if you chose to disengage … (feels like there's a) moral obligation to attend every appointment regardless of the impact on the rest of your life (your carer needs, employment, financial burden of accommodation)*. (ID49, 40 years, f, lung transplant)

## 4 Discussion

In this study, consumers with a range of different health conditions and broad experiences of rehabilitation in Australia shared with us their perspectives about what made it easy and hard to access rehabilitation. Hearing from this diverse group enabled us to identify those common enablers and barriers to access the rehabilitation they needed to support optimal recovery. Although questions about accessing information and the enablers and barriers were asked separately during the focus groups and interviews, the responses highlighted four themes that were foundational to all. These were as follows: service‐centricity of options limits access, access is the patient's responsibility, enabling decision‐making about rehabilitation with appropriate information and provision of a psychologically safe environment. We suggest that these themes should be considered not only to enable greater access to existing services but also in the design and development of new services that may help overcome issues created within the system that can make current offerings not fit for purpose.

Although patients acknowledged that needing to access rehabilitation generally occurred during a stressful time in their life, it was clear that they wanted to be enabled, supported and informed to make decisions that supported their holistic goals, which included accommodating those roles and responsibilities outside of their rehabilitation sessions. Addressing the full range of bio‐psycho‐social health needs was a clear priority for consumers, who wanted information and service offerings that provided flexibility in approach, modality and timing, to willingly accommodate different personal and access needs. Addressing the holistic health needs of patients is a stated priority by the WHO 2023 [3]. Participants felt that their engagement in rehabilitation would be improved if tailored to their situation, considering their needs and preferences (including their health literacy, transport arrangements, financial state, responsibilities and schedule) rather than adopting a ‘one‐size‐fits‐all’ approach. It is widely recognised that successful shared decision‐making requires an interplay between a patient's social, psychological and physical factors that impact on their engagement and adherence to medical rehabilitation programmes [9, 17, 18, 19]. A major barrier to shared decision‐making occurs when generic bench marking is used in goal setting for rehabilitation outcomes, rather than adoption of a personalised approach [20]. Factors beyond physical capacity can include kinesiophobia, fear of reinjury, anxiety and depression, levels of social support and socioeconomic status, and consumers' life roles and family commitments have been linked to motivation to engage and adhere to rehabilitation programmes [17, 19, 21]. Although we identified a need for rehabilitation services to be tailored to consumer's needs, person‐centred goal setting shaped by the healthcare professional can be impacted by their inherent beliefs and attitudes, and organisational limits such as the time and cost of consultations [22].

Participants expressed dissatisfaction and frustration being left with the responsibility to access rehabilitation services when their understanding of clinical situation, rehabilitation needs and available services was low. Barriers that relate to the navigation of the healthcare system have been shown to be modifiable by the healthcare provider or service organisation [23]. A recent scoping review reported that the expertise of patients and their carers' in acquiring knowledge regarding service provision was often underrated by healthcare providers, and as such consumers' input can be disregarded in shared decision‐making consultations [23]. This could be overcome if shared knowledge sharing approaches were adopted from the outset.

The need for provision of quality and appropriate information to consumers about medical rehabilitation options was highlighted in our study and has been reported in other studies to improve engagement and adherence to therapy [9, 17, 18]. In line with the current findings, the major barriers to engaging in rehabilitation after total knee replacement patients were a lack of support from healthcare professionals due to receiving inconsistent or ambiguous information or conflicting information from different specialists [9]. Organisational processes can also impede the sharing of information in a manner to support patients to engage with rehabilitation. There have also been reports that referral pathways for physiotherapy service provision are too cumbersome for healthcare providers, and impact on effective and timely service provision [21]. The Australian Faculty of Rehabilitation Medicine (AFRM) noted, in its 2019 guidelines for inpatient adult rehabilitation, that facilities should develop guideline documentation for outsourcing referrals to appropriate medical rehabilitation service providers if expertise or equipment could not be accessed internally [24]. However, this approach can result in further consumer delays due to additional waiting times with the secondary provider [25].

Having access to information at the right time was deemed important: ill‐timed information has been reported to overburden patients and their carers and impact the effectiveness of the information provided [20]. This can easily occur where a patient's rehabilitative care is supported or delivered by a multidisciplinary team, as this requires complex communication and referral pathways for patients to access and or receive appropriate services [25, 26]. Survivors of stroke have requested information about medical rehabilitation services be provided at different times during their recovery journey, and be provided in different format options, which reflect their changing status and needs during recovery [27]. Others have reported concerns with patients' capacity to comprehend and retain information when provided during times of pain and stress [20]. With the increasing use of digital health platforms in providing patient education, concerns have been raised regarding the content and format of information about rehabilitation services to ensure maximum uptake. In a recent systematic review of qualitative studies regarding the use of virtual education platforms for cardiac rehabilitation, online platforms were found to improve accessibility to information about rehabilitation services and enhance connectivity of support groups, however, can be hampered by a lack of computer literacy or access to appropriate devices or internet connectivity [28]. These challenges can be overcome if considered appropriately during initial interactions between patients and health services, and not be left to the patients to overcome independently when the need for rehabilitation is acute.

In the current study, consumers reported being heard, recognised and respected as crucial factors in accessing, participating and ongoing engagement in rehabilitative services. Indeed, other studies have reported that poor communication across all settings can have substantial impacts on patient satisfaction and programme outcomes [26, 29]. The need for respect and trust with rehabilitation providers has also been reported in other studies, which attributed the interpersonal skills of the healthcare professionals as a key enabler for continued client engagement [7, 30, 31]. Despite the AFRM stipulating the need for a collaborative approach between patients and their rehabilitation teams to establish meaningful and achievable treatment goals [24], many healthcare professionals still lack the capacity to establish clear lines of communication with their patients to enable appropriate and meaningful goal setting, shared decision‐making processes and action planning [22, 32]. This may be particularly the case when the present system (and funding models) focuses on the rehabilitation of ‘primary diagnoses’ as opposed to adopting a more holistic biopsychosocial model of illness and rehabilitation.

### 4.1 Strengths and Limitations

In the current study, we identified barriers and enablers to rehabilitation service access across a wide cross‐section of consumers, with the identified themes having relevance to any healthcare context. Our findings can therefore be used to inform the design of policies and services beyond the Australian healthcare system. This study has provided a rich understanding of consumer's perceived issues to access rehabilitation, from a diverse cohort with respect to age, gender, diagnoses, disability status or identity, rurality and time since, length of and mode of rehabilitative care. People with aphasia and other communication disabilities were included because we designed for and accommodated their needs. Although we were prepared to use healthcare interpreters for people who were not comfortable to participate in English, we did not recruit non‐English speakers; everyone in our study participated in English. We did not collect data on Aboriginal or Torres Strait Islander identity, race or ethnicity more broadly or socioeconomic status, which limits the ability to generalise to other cohorts. A strength of any study that seeks to understand consumer perspectives is the inclusion of carers given their pivotal role in supporting consumer engagement in rehabilitation programmes and services. Although low in number, carers' views informed the findings of this work. We acknowledge that nearly half of our consumers had recently undergone a knee replacement, however, we have included patients with different clinical needs and varied social and demographic backgrounds and as such enabled us to identify themes relevant to a wide cross section of patients. As all participants had engaged with rehabilitation programmes, we cannot comment on the views held by those who chose not to participate in rehabilitation and the reasons behind their decisions. This is an important consideration and may be investigated in future studies. We acknowledge the potential bias inherent in any study that asks for voluntary participation; however, the conversations with participants during the recruitment process highlighted this was not a forum for feedback to the health system, but a study designed to inform the review and design of future rehabilitation services and approaches.

## 5 Conclusions

This study is Part 2 of two papers documenting the perspectives of consumers to support increased access and engagement with rehabilitation services. For consumers, rehabilitation approaches being personalised, shared responsibility for access to services, provision of information to support their decision‐making and feeling psychologically safe were identified as key themes to access rehabilitation. This is consistent with the recent observation made by Eric Wade, a UK‐based Professor of rehabilitation and former Editor‐in‐Chief of the journal *Clinical Rehabilitation*, who wrote in their farewell editorial that rehabilitation is ‘*a way of thinking, not a way of doing*, and that it ought to be *holistic, person‐centred, and concerned about social integration rather than disease or disability*’ [33]. Participants in this study identified their strong desire to be respected as unique individuals with holistic needs, who want to be supported as they access and move through the health system. The information obtained during this study provides key themes with context to help (re)design future services to meet this recommendation and meet consumer needs and expectations more effectively.

## Author Contributions

**Gillian Mason:** investigation, writing–review and editing, formal analysis, data curation, writing–original draft. **Karen Ribbons:** writing–original draft, writing–review and editing. **Lucy Bailey:** project administration. **Adrian O'Malley:** writing–review and editing, formal analysis. **Tracy Ward:** writing–review and editing, formal analysis. **Stephen Ward:** Writing–review and editing, formal analysis. **Michael Pollack:** writing–review and editing, conceptualisation, supervision. **Frederick R. Walker:** conceptualisation, funding acquisition, writing–original draft, writing–review and editing, supervision. **Michael Nilsson:** conceptualisation, funding acquisition, supervision. **Nicolette Hodyl:** investigation, formal analysis, writing–review and editing, supervision.

## Conflicts of Interest

The authors declare no conflicts of interest.

## Supporting information

Supporting information.

## Data Availability

The data are not publicly available due to privacy or ethical restrictions.
