# Supplementary material for: Exploring Consumer Experiences of Barriers and Enablers to Accessing Rehabilitation That Meets Their Needs: The Rehabilitation Choices Study, Part 2—Consumer Perspectives
Source: Health Expect. 2024 Sep 24;27(5):e70035. doi: 10.1111/hex.70035 (PMC11420657; doi:10.1111/hex.70035)
Supplement: Supplementary file 1 — Supporting information. [file HEX-27-e70035-s001.docx]

**Supplementary Material: Additional supporting quotes for each theme.**

|  |  | | |  |  |  |  |
| --- | --- | --- | --- | --- | --- | --- | --- |
| **Theme 1: Service centricity of options limits access** | | | |  |  |  |  |
|  | “I had to start too quickly and before I understood what was wrong with me.” *[ID17, 41y, m, stroke]*  "Rehab was done, because they were done with her, rather than she was actually done. It was only a month and a half into what they said initially was going to be a three-month program, but they said to us ‘…you know over Christmas/New Year the physios and the speech therapists won't be here because they're on holiday.’ So all of a sudden, it was more about the unavailability of the rehab people than it was for the whether (she) was finished with her rehab”. *[ID13, 70y, m, carer of survivor of stroke]*  “…you couldn't choose the time, and when you are needing people to drive you there and you've got people driving, you know, your kids to school that you might have normally done… There is a lot of arranging you have to do, but you have to fit into their schedule.” *[ID50, 46y, f, cardiac event]*  “It was helpful to be able to book session times for rehab activities in advance, be able to plan life around it.” *[ID19, 79y, m,TKR]*  “The logistics of you know, just getting yourself to an appointment for an assessment (that could have been by telehealth), when you can barely walk you know 10 or 15 meters. Catching a two-hour train trip to see a specialist and he gets you in and out in five minutes and you think, ‘well what the hell was that all about?’. And then you come home in pain.” *[ID3, 47y, m, back pain]*  “Mental health…I think it’s the most important thing… if you don’t have that, then you’re not going to have any opportunity to… access anything”. *[ID23, 46y, f, stroke]* | | |  |  |  |  |
|  |  | | |  |  |  |  |
| **Theme 2: Access is the patient’s responsibility** | | | |  |  |  |  |
|  | “No-one helped with organising transport. My husband's blind so he couldn't drive me.” *[ID26, 81y, f, TKR]*  “If someone has communication support needs, involve their carer, spouse or representative - get communication support, rather than just revert to the standard rehab approach because the person hasn't understood (and been involved in goal setting).” *[ID13, m, 70y, carer for survivor of stroke]*  “…for people who don't understand the system or can't communicate quickly (or need accommodations to communicate), if you can't understand and communicate quickly, you can't be included in the planning.” *[ID21, 61y, f, stroke]*  "I can't imagine what the hospital would do if you chose to disengage…moral obligation to attend every appointment regardless of the impact on the rest of your life (carer needs, employment, financial burden of accommodation).” *[ID49, 40y, f, transplant]*  "I don’t understand half of what you’re saying", (someone with no medical background*). [ID23, 48y, f, stroke]*  “Even trying to fill in all those forms to get this funding is very difficult, the average person probably can’t do it.” *[ID2, 67y, f, stroke]*  “…it’s the mental energy you need to wade through the bureaucracy and the system inefficiencies,” *[ID3, 47y, m, back pain]*  "...if you want funding, you have to get your GP to sign off on it, and if they don't fill the form out correctly, then you back there five different times and ...when our energy is a major commodity of ours it's so valuable. You're using all this energy on non-productive things. And so the system is designed for you to fail." *[ID52, 61y, m, stroke]*  "When I've researched it myself I've found I've ended up quite disappointed and it can become very expensive to try and find your way and fail along the way, until you find the right person." *[ID4, 48y, f, chronic inflammatory demyelinating polyneuropathy]*  “It's funny, how the time you need to be psychologically strong to wade through this stuff, is the time that you’re probably at your weakest... you're losing this ability to process things and yet it's never been more important to process things.” *[ID3, 47y, m, back pain]*  “…after that I just sort of felt like I was abandoned, and I just if I wanted more stuff than I had to go and find it myself.” [*ID4, 48y, f, chronic inflammatory demyelinating polyneuropathy]*  “If you had a good starting point, which is a discharge process that actually involved you as a stroke survivor, if you could be involved, to whatever level, and your family or support people, that (your involvement) could grow.” *[ID21, 61y, f, stroke]* | | |  |  |  |  |
| **Theme 3: Enabling decision making about rehabilitation with appropriate information** | | | | |  |  |  |
|  | “Someone phones you when you're not ready to receive information, without warning.  I could be at the supermarket or surrounded by family at the time and not able to concentrate  on the conversation.” *[ID48, 48y, f, fractured wrist]*  "It’s the filtering. You have so much information to get to what you want to know about yourself.  There is so much information out there and haven't got the time, have you got the energy?  When can you do it?" *[ID22, 52y, m, stroke]*  “[forms for funding support] they're not clearly written; you don't quite understand what they really  asking you for anyway*.” [ID2, 61y, f, stroke]*  “I had left work, so I had all this time, which I think time was the biggest factor to be able to put this  all into place*.” [ID33, 62y, f, TKR]*  “I had no idea how to choose, (which rehab hospital) so I was just pointing off the list,  like ‘Oh, I’ll have that one!’ I was given access to a list of names of people who provide  services. But I didn't know [anything about] them, so it was more luck than good management  who I got to choose…there wasn't anything that I…could access, to say oh this person's a really  good one, or I had bad experiences with this person”. *[ID13, 71y, m, carer of survivor of stroke]*  "It's not only having the financial ability to access therapy services, rehab, it's actually about knowing  that you, you can use that money that you've got wisely with somebody who's skilled enough to  do the job that you're needing because, otherwise,  … you're not getting the best use of your therapy time…bang for buck *[ID22, 52y, m, stroke]*  “I feel there is a great lack of communication right along the lines. unless there's someone  who's actually experienced things and you've spoken to them, you would not have any idea  (what to expect).” *[ID41, 79y, f, TKR]*  “The information was for people who had a heart attack from bad health, bad lifestyle choices  and the information I found didn't necessarily relate to me.” *[ID50, 46y, f, cardiac]*  "I found if you cannot communicate clearly, then you've got no hope...I still have trouble  trying to communicate over the phone". *[ID2, 61y, f, stroke]*  "They expect people who like with…my conditions or any chronic pain conditions  (to understand everything quickly), we all suffer from brain fog…you can be as intelligent  and capable, as can be, but how you meant to navigate through all this when your brain isn't  allowing you to function properly?" *[ID4, 48y, f, chronic inflammatory demyelinating polyneuropathy]*  “If they start to tell you all the things that you're going to go through…  I'd rather just jump into it like having a baby. I don't want anyone to tell me how bad it's  going to be.” *[ID24, 56y, f, TKR]*  "One of the things I'm concerned about is because of COVID, a lot of therapy has become digital.  Like telehealth. There have been occasions where my level of distress has escalated massively  because I’m battling with technology, and it's not straightforward. Telehealth is fantastic,  but I sometimes think that due diligence about the user's capacity isn't always taken into  account." *[ID21,61y, f, stroke]* | | | |  |  |  |
| **Theme 4: Provision of a psychological safe environment** | | | | | | | |
|  | “For me... it's the level of empathy of the professional...wanting to see a professional because you know  them and you like them, and you trust them... the levels of empathy we get with professionals vary a lot...  whether that's through just professionalism or it's just a natural personality trait... that sort of helps motivate  me to get to see them.” [ID3, 47y, m, back pain]  “I was not able to communicate anything about myself as a person and they didn't ask my wife either.  Maybe they wouldn't have asked at all. Felt like nothing was designed for me.” *[ID17, 41y, m, stroke]*  “I felt like a medical record number, not a person.” *[ID17, 41y, m, stroke]*  “The assumptions that can very easily be made about we fit into this category or that category, or we can  be serviced this way or that way or access it is this way or that way, but the unique identity of the individual  is forgotten, too easily forgotten.” [ID*52, 61y, m, stroke]*  “The (rehab) team didn't really treat you like you knew anything...they kind of don't give a rat's if you  know (things about your condition)…he didn't really seem to care at all about me because he wasn't  interested in anything I had to say," *[ID49, 40y, f, transplant]*  “…because I was young (compared with others in the service)…it was really hard to engage with others,  because they all thought, well, at 36 you know, you’ll be right! It felt very  isolating to be so young.” *[ID50, 46y, f, cardiac]*  “It’s isolating being at the ‘centre’ of all the care, when you’re not actually involved in the decision-making  about any of it.” [ID4, 48y, f, chronic inflammatory demyelinating polyneuropathy]  "Where they talk about patient-centred care it's bullshit. They just do it, but if they actually were really doing  patient-centred care, or family-centred involvement, a good discharge plan would have the opportunity to flesh  out what a person was needing and wanting. And then, where they could go to get it. But invariably, your  discharge plan is your discharge letter, quite frankly." *[ID21, 61y, f, stroke]*  “Not being connected to a clinician...that they’re not interested in your recovery… it's just an appointment,  for them, it’s just a thing in their diary. And this (affects how easy it is to engage in rehab) because it's the  case that you can’t move on, you see the glass half empty, so you think, ‘No. I’m not going to do it.’  Recovery depends on the positive or negative experience that you have.” *[ID23, 46y, f, stroke]*  “It takes a lot for us to get ourselves, whether it's physically or mentally, to one of these services and so,  once we get that we want to feel validated....  to be treated with respect and validation.” *[ID4, 48y, f, chronic inflammatory demyelinating polyneuropathy]*  "Yes, if the empathy is not there. Or if it is below standard, there's a good chance that you won't hear  the points of significance to you…you mightn’t even be aware that it’s really significant it might be just  a throwaway line, but it can be really, really important and you need that empathy for the professional  opinion to come to you." *[ID52, 61y, m, stroke]* | | | | | |  |
